# Supplementary material for: From sequence to enzyme mechanism using multi-label machine learning
Source: BMC Bioinformatics. 2014 May 19;15:150. doi: 10.1186/1471-2105-15-150 (PMC4229970; doi:10.1186/1471-2105-15-150)
Supplement: Additional file 2 — Java code of ml2db. Additional file ml2db_code.tar.gz contains the Java source code to run the multi-label machine learning experiments and save the results to database. The code’s Javadoc is included. [file 1471-2105-15-150-S2.zip › additional file 2/ml2db/utils/doc/uk/ac/standrews/utils/main/database/DbUtils.html]

DbUtils


JavaScript is disabled on your browser.


- Overview
- Package
- Class
- Use
- Tree
- Deprecated
- Index
- Help

- Prev Class
- Next Class

- Frames
- No Frames

- All Classes

- Summary:
- Nested |
- Field |
- Constr |
- Method

- Detail:
- Field |
- Constr |
- Method


uk.ac.standrews.utils.main.database

## Class DbUtils

- java.lang.Object
- - uk.ac.standrews.utils.main.database.DbUtils

- ---

    

  ```
  public class DbUtils
  extends java.lang.Object
  ```

  Database utilities (to create sql code etc.)

  Version:
  :   20061125

  Author:
  :   Luna

- - ### Field Summary

    Fields

    | Modifier and Type | Field and Description |
    | --- | --- |
    | `static java.lang.String` | `AUTO_INCREMENT_SQL_DATATYPE` |
    | `static java.lang.String` | `BOOLEAN_SQL_DATATYPE` |
    | `static java.lang.String` | `CHAR_2_SQL_DATATYPE` |
    | `static java.lang.String` | `CHAR_4_SQL_DATATYPE` |
    | `static java.lang.String` | `CHAR_6_SQL_DATATYPE` |
    | `static java.lang.String` | `DOUBLE_QUOTE_CHARACTER` |
    | `static java.lang.String` | `DOUBLE_SQL_DATATYPE` |
    | `static java.lang.String` | `END_TIMESTAMP` |
    | `static java.lang.String` | `ID` |
    | `static java.lang.String` | `INTEGER_SQL_DATATYPE` |
    | `static java.lang.String` | `LONG_TEXT_SQL_DATATYPE` |
    | `static long` | `MAX_FILE_SIZE` maximum size of file that can be stored in database field text ( 1,000,000 bytes ~= 1 Mb) |
    | `static java.lang.String` | `REVERSED_PRIME_CHARACTER` |
    | `static java.lang.String` | `SINGLE_QUOTE_CHARACTER` |
    | `static java.lang.String` | `START_TIMESTAMP` |
    | `static java.lang.String` | `TEXT_SQL_DATATYPE` |
    | `static java.lang.String` | `TIMESTAMP` |
    | `static java.lang.String` | `TIMESTAMP_DOWNLOADED` |
    | `static java.lang.String` | `TIMESTAMP_SQL_DATATYPE` |
    | `static java.lang.String` | `VARCHAR_16_SQL_DATATYPE` |
    | `static java.lang.String` | `VARCHAR_255_SQL_DATATYPE` |
    | `static java.lang.String` | `VARCHAR_26_SQL_DATATYPE` |
    | `static java.lang.String` | `VARCHAR_90_SQL_DATATYPE` |
  - ### Constructor Summary

    Constructors

    Constructor and Description

    `DbUtils()`
  - ### Method Summary

    Methods

    | Modifier and Type | Method and Description |
    | --- | --- |
    | `static boolean` | `elementExistsInTable(java.lang.String table, java.lang.String column, java.lang.String element, DbConn dbConn)` Get if an element exists in a table column |
    | `static boolean` | `elementsExistsInTable(java.lang.String table, java.lang.String column1, java.lang.String element1, java.lang.String column2, java.lang.String element2, DbConn dbConn)` Get if two elements exists in the same table row |
    | `static boolean` | `existsInTable(java.lang.String query, DbConn dbConn)` |
    | `static int` | `getBasicTypeOfDataColumn(java.util.ArrayList<java.lang.String> list)` Given a list of strings, it returns a generic data type for the database column that could best contain them: INTEGER, DOUBLE, STRING If the strings passes a parseInteger: type = INTEGER if the strings contain digits, 'E' (for exponential), dot, plus, minus: type = DOUBLE If the strings contain letters or other characters: type = STRING Note: If the string contains only digits, but also intervening white spaces: type = STRING |
    | `static java.util.ArrayList<java.util.ArrayList<java.lang.String>>` | `getColumnsfrom2DArray(java.lang.String[][] array)` Takes a 2 dimensional array of data and returns the columns (as an array list of array lists) |
    | `static java.lang.String` | `getDataTypeForAnArray(java.util.ArrayList<java.lang.String> list)` Guesses from data type and length the appropriate SQL type of a column to contain the data in the array |
    | `static TableColumn` | `getEndTimeColumn()` A table column to store a timestamp for the end of an experiment |
    | `static TableColumn` | `getIdColumn()` get the definition for an "id" column of type auto-increment (which is primary key) |
    | `static java.lang.String` | `getListOfValuesString(java.util.Vector<java.lang.String> listOfValues, java.lang.String quotationCharacter)` Transforms a list of values into a comma separated list |
    | `static java.sql.ResultSet` | `getResultSet(java.lang.String sql, DbConn dbConn)` Executes an sql query returning a result set |
    | `static java.util.Vector<java.lang.String>` | `getResultSetColumnAsVector(java.sql.ResultSet rs, int column)` Takes a resultSet and returns an array of Strings |
    | `static TableColumn` | `getStartTimeColumn()` A table column to store a timestamp for the beginning of an experiment |
    | `static TableColumn` | `getTimeDownloadedColumn()` |
    | `static TableColumn` | `getTimestampColumn()` |
    | `static boolean` | `insert(DbConn conn, java.lang.String insertSql)` Executes sql insert and returns whether the insert was successful. |
    | `static void` | `main(java.lang.String[] args)` |
    | `static void` | `printResultsSet(java.sql.ResultSet results)` Prints the results of a query |
    | `static java.lang.String` | `resultsSetToString(java.sql.ResultSet results)` Generates a string for the results of a query |
    | `static boolean` | `similarElementExistsInTable(java.lang.String table, java.lang.String column, java.lang.String element, DbConn dbConn)` |

    - ### Methods inherited from class java.lang.Object

      `equals, getClass, hashCode, notify, notifyAll, toString, wait, wait, wait`

- - ### Field Detail


    - #### AUTO\_INCREMENT\_SQL\_DATATYPE

      ```
      public static final java.lang.String AUTO_INCREMENT_SQL_DATATYPE
      ```

      See Also:
      :   Constant Field Values


    - #### BOOLEAN\_SQL\_DATATYPE

      ```
      public static final java.lang.String BOOLEAN_SQL_DATATYPE
      ```

      See Also:
      :   Constant Field Values


    - #### CHAR\_2\_SQL\_DATATYPE

      ```
      public static final java.lang.String CHAR_2_SQL_DATATYPE
      ```

      See Also:
      :   Constant Field Values


    - #### CHAR\_4\_SQL\_DATATYPE

      ```
      public static final java.lang.String CHAR_4_SQL_DATATYPE
      ```

      See Also:
      :   Constant Field Values


    - #### DOUBLE\_SQL\_DATATYPE

      ```
      public static final java.lang.String DOUBLE_SQL_DATATYPE
      ```

      See Also:
      :   Constant Field Values


    - #### END\_TIMESTAMP

      ```
      public static final java.lang.String END_TIMESTAMP
      ```

      See Also:
      :   Constant Field Values


    - #### ID

      ```
      public static final java.lang.String ID
      ```

      See Also:
      :   Constant Field Values


    - #### TIMESTAMP

      ```
      public static final java.lang.String TIMESTAMP
      ```

      See Also:
      :   Constant Field Values


    - #### INTEGER\_SQL\_DATATYPE

      ```
      public static final java.lang.String INTEGER_SQL_DATATYPE
      ```

      See Also:
      :   Constant Field Values


    - #### START\_TIMESTAMP

      ```
      public static final java.lang.String START_TIMESTAMP
      ```

      See Also:
      :   Constant Field Values


    - #### TIMESTAMP\_DOWNLOADED

      ```
      public static final java.lang.String TIMESTAMP_DOWNLOADED
      ```

      See Also:
      :   Constant Field Values


    - #### TIMESTAMP\_SQL\_DATATYPE

      ```
      public static final java.lang.String TIMESTAMP_SQL_DATATYPE
      ```

      See Also:
      :   Constant Field Values


    - #### VARCHAR\_90\_SQL\_DATATYPE

      ```
      public static final java.lang.String VARCHAR_90_SQL_DATATYPE
      ```

      See Also:
      :   Constant Field Values


    - #### VARCHAR\_255\_SQL\_DATATYPE

      ```
      public static final java.lang.String VARCHAR_255_SQL_DATATYPE
      ```

      See Also:
      :   Constant Field Values


    - #### TEXT\_SQL\_DATATYPE

      ```
      public static final java.lang.String TEXT_SQL_DATATYPE
      ```

      See Also:
      :   Constant Field Values


    - #### LONG\_TEXT\_SQL\_DATATYPE

      ```
      public static final java.lang.String LONG_TEXT_SQL_DATATYPE
      ```

      See Also:
      :   Constant Field Values


    - #### DOUBLE\_QUOTE\_CHARACTER

      ```
      public static final java.lang.String DOUBLE_QUOTE_CHARACTER
      ```

      See Also:
      :   Constant Field Values


    - #### SINGLE\_QUOTE\_CHARACTER

      ```
      public static final java.lang.String SINGLE_QUOTE_CHARACTER
      ```

      See Also:
      :   Constant Field Values


    - #### REVERSED\_PRIME\_CHARACTER

      ```
      public static final java.lang.String REVERSED_PRIME_CHARACTER
      ```

      See Also:
      :   Constant Field Values


    - #### CHAR\_6\_SQL\_DATATYPE

      ```
      public static final java.lang.String CHAR_6_SQL_DATATYPE
      ```

      See Also:
      :   Constant Field Values


    - #### VARCHAR\_26\_SQL\_DATATYPE

      ```
      public static final java.lang.String VARCHAR_26_SQL_DATATYPE
      ```

      See Also:
      :   Constant Field Values


    - #### VARCHAR\_16\_SQL\_DATATYPE

      ```
      public static final java.lang.String VARCHAR_16_SQL_DATATYPE
      ```

      See Also:
      :   Constant Field Values


    - #### MAX\_FILE\_SIZE

      ```
      public static final long MAX_FILE_SIZE
      ```

      maximum size of file that can be stored in database field text ( 1,000,000 bytes ~= 1 Mb)

      See Also:
      :   Constant Field Values
  - ### Constructor Detail


    - #### DbUtils

      ```
      public DbUtils()
      ```
  - ### Method Detail


    - #### elementExistsInTable

      ```
      public static boolean elementExistsInTable(java.lang.String table,
                                 java.lang.String column,
                                 java.lang.String element,
                                 DbConn dbConn)
      ```

      Get if an element exists in a table column

      Parameters:
      :   `table` - table name
      :   `column` - column name
      :   `element` - the desired string
      :   `dbConn` - the database connection

      Returns:
      :   true if the element exists in the database table


    - #### elementsExistsInTable

      ```
      public static boolean elementsExistsInTable(java.lang.String table,
                                  java.lang.String column1,
                                  java.lang.String element1,
                                  java.lang.String column2,
                                  java.lang.String element2,
                                  DbConn dbConn)
      ```

      Get if two elements exists in the same table row

      Parameters:
      :   `table` - table name
      :   `column1` - column1 name
      :   `element1` - the desired string in column 1
      :   `column2` - column2 name
      :   `element2` - the desired string in column 2
      :   `dbConn` - the database connection

      Returns:
      :   true if the element exists in the database table


    - #### existsInTable

      ```
      public static boolean existsInTable(java.lang.String query,
                          DbConn dbConn)
      ```


    - #### getBasicTypeOfDataColumn

      ```
      public static int getBasicTypeOfDataColumn(java.util.ArrayList<java.lang.String> list)
      ```

      Given a list of strings, it returns a generic data type for the database
      column that could best contain them: INTEGER, DOUBLE, STRING
      If the strings passes a parseInteger: type = INTEGER if the strings
      contain digits, 'E' (for exponential), dot, plus, minus: type = DOUBLE If
      the strings contain letters or other characters: type = STRING Note: If
      the string contains only digits, but also intervening white spaces: type
      = STRING

      Parameters:
      :   `list` - a list of strings (data)

      Returns:
      :   the basic datatype (INTEGER, DOUBLE, STRING)


    - #### getColumnsfrom2DArray

      ```
      public static java.util.ArrayList<java.util.ArrayList<java.lang.String>> getColumnsfrom2DArray(java.lang.String[][] array)
      ```

      Takes a 2 dimensional array of data and returns the columns (as an array
      list of array lists)

      Parameters:
      :   `array` - a 2 dimentional array containing data

      Returns:
      :   a list containing one array list per column of data


    - #### getDataTypeForAnArray

      ```
      public static java.lang.String getDataTypeForAnArray(java.util.ArrayList<java.lang.String> list)
      ```

      Guesses from data type and length the appropriate SQL type of a column to
      contain the data in the array

      Parameters:
      :   `list` - an array of strings

      Returns:
      :   the SQL type of a column for the data in the array


    - #### getIdColumn

      ```
      public static TableColumn getIdColumn()
      ```

      get the definition for an "id" column of type auto-increment (which is
      primary key)


    - #### getListOfValuesString

      ```
      public static java.lang.String getListOfValuesString(java.util.Vector<java.lang.String> listOfValues,
                                           java.lang.String quotationCharacter)
      ```

      Transforms a list of values into a comma separated list

      Parameters:
      :   `listOfValues` - e.g. [a, b, c]
      :   `the` - quotation character to surround the values (usually double
          quotes or apex)

      Returns:
      :   a comma separated list, for example (if the quotation character
          is an apex): 'a', 'b', 'c'


    - #### getResultSet

      ```
      public static java.sql.ResultSet getResultSet(java.lang.String sql,
                                    DbConn dbConn)
      ```

      Executes an sql query returning a result set

      Parameters:
      :   `sql` - an sql query

      Returns:
      :   a resultSet

      Throws:
      :   `java.sql.SQLException`


    - #### getResultSetColumnAsVector

      ```
      public static java.util.Vector<java.lang.String> getResultSetColumnAsVector(java.sql.ResultSet rs,
                                                                  int column)
      ```

      Takes a resultSet and returns an array of Strings

      Parameters:
      :   `rs` - a ResultSet
      :   `column` - the column number (1 = the first column)

      Returns:
      :   a `Vector` with the query results


    - #### getTimeDownloadedColumn

      ```
      public static TableColumn getTimeDownloadedColumn()
      ```


    - #### getTimestampColumn

      ```
      public static TableColumn getTimestampColumn()
      ```


    - #### insert

      ```
      public static boolean insert(DbConn conn,
                   java.lang.String insertSql)
      ```

      Executes sql insert and returns whether the insert was successful.

      Parameters:
      :   `conn` -
      :   `insertSql` -

      Returns:
      :   true if the insert was successful


    - #### main

      ```
      public static void main(java.lang.String[] args)
      ```


    - #### printResultsSet

      ```
      public static void printResultsSet(java.sql.ResultSet results)
      ```

      Prints the results of a query

      Parameters:
      :   `results` - a query results set


    - #### resultsSetToString

      ```
      public static java.lang.String resultsSetToString(java.sql.ResultSet results)
      ```

      Generates a string for the results of a query

      Parameters:
      :   `results` - a query results set


    - #### similarElementExistsInTable

      ```
      public static boolean similarElementExistsInTable(java.lang.String table,
                                        java.lang.String column,
                                        java.lang.String element,
                                        DbConn dbConn)
      ```


    - #### getStartTimeColumn

      ```
      public static TableColumn getStartTimeColumn()
      ```

      A table column to store a timestamp for the beginning of an experiment

      Returns:
      :   A table column to store a timestamp for the beginning of an experiment


    - #### getEndTimeColumn

      ```
      public static TableColumn getEndTimeColumn()
      ```

      A table column to store a timestamp for the end of an experiment

      Returns:
      :   A table column to store a timestamp for the end of an experiment


- Overview
- Package
- Class
- Use
- Tree
- Deprecated
- Index
- Help

- Prev Class
- Next Class

- Frames
- No Frames

- All Classes

- Summary:
- Nested |
- Field |
- Constr |
- Method

- Detail:
- Field |
- Constr |
- Method
